# Periplasmic Bacterial Biomineralization of Copper Sulfide Nanoparticles

**DOI:** 10.1002/advs.202203444

**Published:** 2022-08-17

**Authors:** Yeseul Park, Zohar Eyal, Péter Pekker, Daniel M. Chevrier, Christopher T. Lefèvre, Pascal Arnoux, Jean Armengaud, Caroline L. Monteil, Assaf Gal, Mihály Pósfai, Damien Faivre

**Affiliations:** ^1^ Aix‐Marseille University French Alternative Energies and Atomic Energy Commission (CEA) French National Center for Scientific Research (CNRS) UMR7265 Institute of Biosciences and Biotechnologies of Aix‐Marseille (BIAM) Saint‐Paul‐lez‐Durance 13108 France; ^2^ Department of Plant and Environmental Sciences Weizmann Institute of Science Rehovot 7610001 Israel; ^3^ Nanolab, Research Institute of Biomolecular and Chemical Engineering University of Pannonia Egyetem st. 10 Veszprém 8200 Hungary; ^4^ Medicines and Healthcare Technologies Department (DMTS) University of Paris‐Saclay French Alternative Energies and Atomic Energy Commission (CEA) National Research Institute for Agriculture, Food and the Environment (INRAE) Pharmacology and Immunoanalysis unit (SPI) Bagnols‐sur‐Cèze 30200 France; ^5^ ELKH‐PE Environmental Mineralogy Research Group Egyetem st. 10 Veszprém 8200 Hungary

**Keywords:** biologically‐controlled biomineralization, copper sulfide, cryo‐electron tomography, intracellular biomineralization, magnetotactic bacteria, proteomics

## Abstract

Metal sulfides are a common group of extracellular bacterial biominerals. However, only a few cases of intracellular biomineralization are reported in this group, mostly limited to greigite (Fe_3_S_4_) in magnetotactic bacteria. Here, a previously unknown periplasmic biomineralization of copper sulfide produced by the magnetotactic bacterium *Desulfamplus magnetovallimortis* strain BW‐1, a species known to mineralize greigite (Fe_3_S_4_) and magnetite (Fe_3_O_4_) in the cytoplasm is reported. BW‐1 produces hundreds of spherical nanoparticles, composed of 1–2 nm substructures of a poorly crystalline hexagonal copper sulfide structure that remains in a thermodynamically unstable state. The particles appear to be surrounded by an organic matrix as found from staining and electron microscopy inspection. Differential proteomics suggests that periplasmic proteins, such as a DegP‐like protein and a heavy metal‐binding protein, could be involved in this biomineralization process. The unexpected periplasmic formation of copper sulfide nanoparticles in BW‐1 reveals previously unknown possibilities for intracellular biomineralization that involves intriguing biological control and holds promise for biological metal recovery in times of copper shortage.

## Graphical abstract

Some bacteria have the capability of forming inorganic nanoparticles in a process called biomineralization. A magnetotactic bacterium is shown here, that can precipitate an unknown type of intracellular copper‐based nanoparticles in the periplasm. The biomineral shows peculiar nanometric sub‐structures, and a potential organic envelope surrounding the particles. The protein analysis points toward a new biomineralization mechanism involved in copper detoxification.

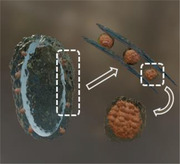

## 1 Introduction

Various organisms produce inorganic minerals through a process called biomineralization.^[^ ^1^ ^]^ According to Lowenstam's original definition, biomineralization can be classified into biologically‐induced and biologically‐controlled processes.^[^ ^1^ ^]^ The first case mainly refers to interactions between metabolic byproducts and environments and leads to particles with heterogeneous properties.^[^ ^2^ ^]^ The latter occurs under strict cellular control.^[^ ^3^ ^]^ Such biomineralization processes often lead to materials with highly controlled particle dimension and organization, defined location of nucleation, and compartmentalization, thus yielding uniform nano‐ and microstructured materials with potential for numerous applications.^[^ ^4^ ^]^

Microbial biomineralization contributes to the formation of both extracellular and intracellular biominerals.^[^ ^5^ ^]^ In particular, sulfate‐reducing bacteria play an important role in the environment, by producing diverse extracellular biominerals, especially metal sulfides.^[^ ^6^ ^]^ In contrast, only a few cases of biologically‐controlled intracellular metal sulfide biomineralization have been identified in microorganisms,^[^ ^7^ ^]^ representatively CdS,^[^ ^8^ ^]^ and Fe_3_S_4_.^[^ ^9^, ^10^, ^11^ ^]^ Microorganisms with the strictest control over biomineral structures are known to be magnetotactic bacteria, which form magnetosomes, organelles consisting of either greigite (Fe_3_S_4_) or magnetite (Fe_3_O_4_) crystals in lipidic vesicles localized within the cytoplasm.^[^ ^12^ ^]^ Periplasmic biomineralization reported in bacteria commonly appears in the form of encrustation within the periplasm.^[^ ^13^, ^14^, ^15^ ^]^ Such processes are understood to fall under the category of biologically‐induced biomineralization,^[^ ^16^ ^]^ although a study suggested that the process could involve biomolecules.^[^ ^17^ ^]^

In this study, we demonstrate that the periplasmic biomineralization of copper sulfide nanoparticles we discovered is a biologically‐controlled process. In particular, we report the biomineralization of copper sulfide in the magnetotactic bacterium *Desulfamplus magnetovallimortis* strain BW‐1.^[^ ^18^ ^]^ This bacterium produces hundreds of spherical nanoparticles of about 70 nm within the periplasmic space. Each particle is composed of 1–2 nm‐sized building blocks with crystallographic domains of hexagonal CuS phases that are thermodynamically unstable and appears surrounded by an organic envelope. Our differential proteomic analysis points toward putative periplasmic proteins that could be responsible for the biomineralizing process.

## 2 Results and Discussion

**Figure 1:**
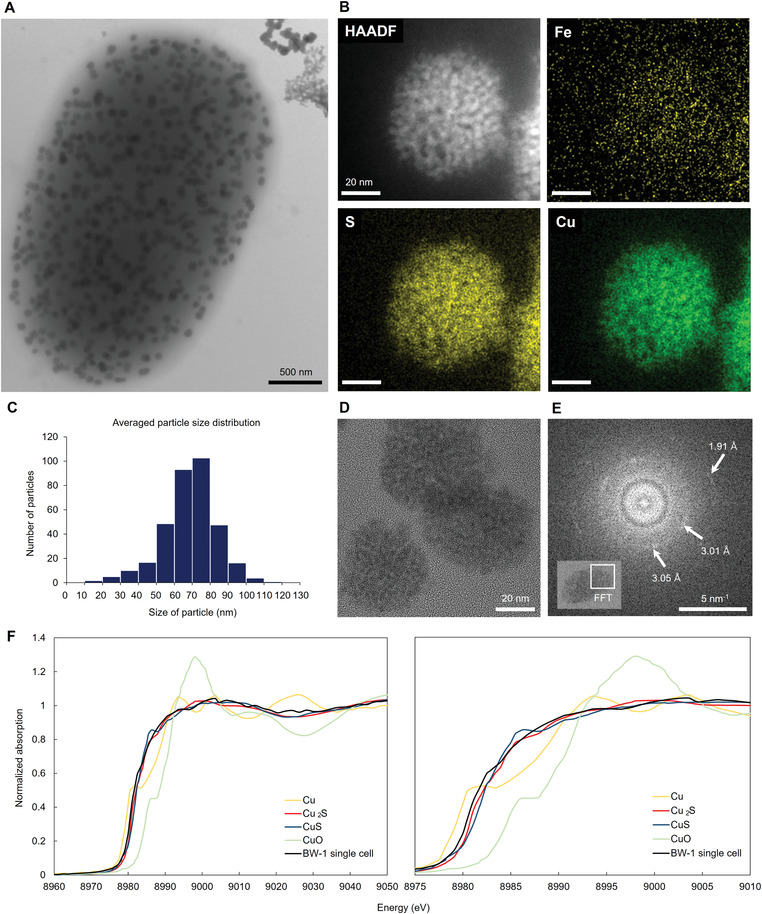
Morphological and chemical analysis of copper sulfide nanoparticles produced by BW‐1. A) A BW‐1 cell filled with intracellular nanoparticles, showing features distinct from extracellular precipitates, as imaged by TEM. B) HAADF STEM image showing a single copper sulfide nanoparticle in a BW‐1 cell and STEM0EDS element maps of Fe, S, and Cu, respectively. C) Size distribution of particles (10 bacteria). D) HRTEM image of copper sulfide particles composed of 1–2 nm‐sized substructures. E) Fourier transform of the boxed area in the inset, showing two faint rings and some diffuse spots. F) Normalized Cu K‐edge XAS spectra from an entire single cell presented in Figure S3, Supporting Information together with reference materials. The left spectrum covers the energy range 8960–9050 eV and shows the first part of the EXAFS oscillation, and the right spectrum is an inset covering the energy range 8975–9010 eV to show the pre‐edge and rising edge features in more detail.

The bacterial strain we used is a sulfate‐reducer and the only isolated case among greigite‐producing magnetotactic bacteria. This bacterium is capable of biomineralizing both magnetite and greigite.^[^ ^18^, ^19^ ^]^ As environmental greigite producers commonly contain significant amount of copper in their biomineral,^[^ ^20^, ^21^ ^]^ we hypothesized that greigite biomineralization by BW‐1 could be influenced by copper. We thus increased copper concentration in the medium up to 13.9 µm. In this condition, we discovered that BW‐1 produced hundreds of nanoparticles inside the cell body, with a peculiar localization, arrangement, and morphology, while magnetite and greigite magnetosomes remained absent. First, the particles show different morphological features than magnetosome particles or extracellular precipitates (**Figure** **1**A, Figure S1, Supporting Information). The nanoparticles appear spherical and separated from each other, with a narrow size distribution (69 ± 15 nm). The number of particles per cell is 344 ± 76 (Figure 1C). These particles are absent at the low Cu concentration of the culture medium (0.73 µm).

As demonstrated in Figure 1B (also Figures S2E,F, Supporting Information), the nanoparticles are mainly composed of copper and sulfur. The distribution of both elements is similar in each particle and there is no noticeable accumulation of these elements apart from the particles (Figures S2B, S3D, S2E, Supporting Information). Neither magnetosome particles, nor iron and phosphorus‐rich granules that are frequently observed in anaerobic bacteria, such as ferrosomes^[^ ^22^ ^]^ were observed, apart from the copper sulfide particles. Quantitative energy dispersive X‐ray spectroscopy (EDS) analysis revealed an averaged elemental ratio of Cu/S of 1.39 ± 0.15 (Figure S2C, Supporting Information). The copper sulfide crystal phase with the closest Cu/S ratio is hexagonal copper sulfide spionkopite (Cu_39_S_28_, Cu/S ratio 1.39).

Looking in more detail with high‐resolution TEM (HRTEM), the particles remarkably consist of 1–2 nm‐sized substructures, which are in the similar size range as CdS‐capping agent complexes reported in yeasts^[^ ^23^ ^]^ (Figure 1D, Figure S2D, Supporting Information). Fast Fourier transforms (FFTs) of HRTEM images of single particles show two faint rings with several diffuse reflections within the rings (Figure 1E). This indicates that the individual crystallites are not coaligned, and are nm‐sized. Two d‐spacing values can be measured in the FFT, ranging between 3.04 and 3.08 Å (the inner ring) and between 1.86 and 1.90 Å (the outer ring). These values match those of the two most intense reflections of hexagonal copper sulfides, including covellite, spionkopite, and yarrowite (in covellite 1.897 Å corresponds to d(110) and 3.048 Å to d(102)).^[^ ^24^, ^25^ ^]^ One of the strongest reflections of covellite at 2.7 Å (006) did not appear.^[^ ^26^ ^]^ Only one similar value exists in chalcocite (3.06 Å for d(101)). On the other hand, the diffraction data do not match spacings in other common copper sulfide minerals that have cubic close‐packing of sulfur (digenite, geerite, anilite). Accordingly, both elemental composition and structural data suggest that the constituent crystallites of the spherical particles have hexagonal packing of sulfur layers in the short range.

For further information, we collected Cu K‐edge X‐ray absorption spectroscopy (XAS) data on individual BW‐1 cells to examine them from the local structural perspective. Based on the pre‐edge (1s→3d) and rising edge (1s→4p) near‐edge features, the XAS spectrum of a BW‐1 cell (Figure 1F, Figure S3F, Supporting Information) shows features more similar to Cu_2_S than CuS, which also indicates less Cu^2+^ in the biomineral composition. Specifically, the characteristic rising edge feature from CuS (≈8986 eV) and more prominent pre‐edge feature (spin‐forbidden 1s→3d^9^ transition for Cu^2+^ centers) are absent in the BW‐1 sample.^[^ ^27^ ^]^ Moreover, Cu_2_S and the cell sample have similar absorption edge positions and relatively weak near‐edge features. Combining TEM and XAS data, the observed features of the copper sulfide particles are consistent with the properties of a “primitive copper sulfide precipitate” described by Pattrick et al.;^[^ ^28^ ^]^ accordingly, we conclude that the newly‐discovered biomineral has a covellite‐type short‐range order, and contains Cu(I), mostly in trigonal coordination. Such a “primitive structure” of the biomineral is supposed to change into a more stable state with time. However, the copper sulfide nanoparticles produced by BW‐1 remain in the described metastable state. Accordingly, the structural feature suggests the involvement of biological control in the formation and conservation of the particles.

**Figure 2:**
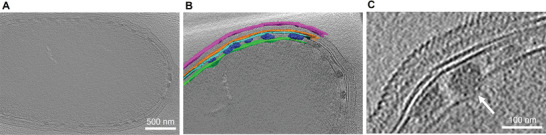
Location of intracellular copper sulfide nanoparticles in BW‐1. A) Cryogenic electron microscopy (cryo‐EM) tomographic slice of a BW‐1 cell. B) Volume rendering of the intracellular particles and four cell membranes (magenta: S‐layer, orange: outer membrane, cyan: peptidoglycan layer and green: plasma membrane) overlapped on a cryo‐EM tomographic slice. C) Cryo‐EM tomographic slice focused on copper sulfide nanoparticles. A white arrow indicates an expanded part of the periplasmic space due to the presence of a copper sulfide particle.

In order to elucidate the cellular localization of the mineral particles, we performed cryo‐electron tomography (cryo‐ET) of hydrated cells to investigate the native‐state cellular organization of the bacteria. A slice in the tomogram reconstruction in **Figure** **2**A and the 3D volume rendering in Figure 2B and Movie S1, Supporting Information clearly indicate that the nanoparticles are localized inside the periplasmic space. Some particles locally distort the inner membrane and extend the surrounding periplasmic area (Figure 2C, Figure S4, Supporting Information). This distortion is against the observation that the periplasmic space of gram‐negative bacteria cannot be artificially extended because of bacterial homeostasis to control the width of the periplasm.^[^ ^29^ ^]^ The expansion of the periplasmic space caused by enlargement of the particle could be interpreted either as the result of cellular adjustment involving hydrolysis of peptidoglycan layers that sustain the periplasmic space or as the result of disruption in homeostasis within the periplasm by biomineralization processes.

**Figure 3:**
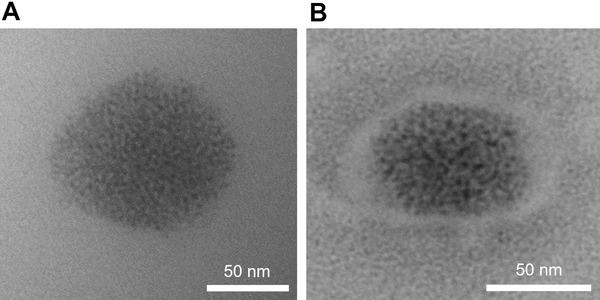
Particles extracted from cells using a cell disruptor and deposited onto TEM grids. A) Particle before staining. B) Particle showing a potential macromolecular complex after a staining with sodium tungstate 2% w/v.

As biologically‐controlled mineralization typically occurs within an organic matrix,^[^ ^3^ ^]^ we searched for such a configuration. After rupturing the BW‐1 cells, we deposited the particles onto a TEM grid. Comparing unstained and stained particles (**Figure** **3**A, Figure S5A, Supporting Information), a pattern of an encompassing substance was observed on every particle (Figure 3B, Figure S5B, Supporting Information). Given the absence of a distinct phosphorus signal around the particle (Figure S2B, Supporting Information), it may not be a typical lipidic membrane but a different type of organic matrix composed of a macromolecular complex. The biomineral particles possibly remain separated from each other within the periplasm thanks to this organic matrix. This distribution of biomineral is observed at an even higher concentration of Cu (147 µm) (Figure S6A, Supporting Information).

The biomineralization we observe is both a metal‐specific and an organism‐specific process. The formation of metal sulfide biominerals was indeed tested with different metal ions: Zn, Ni, and Co (at the concentration of 147 µm). No intracellular biomineral was observed in these cases (Figures S6B–D, Supporting Information respectively). The copper sulfide biomineralization was also tested in the culture of *Desulfovibrio magneticus* strain RS‐1,^[^ ^22^ ^]^ the most studied MTB affiliated to the same class as BW‐1 (at the copper concentration of 13.9 µm). RS‐1 does not produce any intracellular biomineral in the periplasm (Figure S6E, Supporting Information). These results indicate that the observed Cu‐sulfide biomineralization in BW‐1 is both metal‐specific and organism‐specific. Accordingly, the result also suggests that the observed biomineralization is not a simple biologically‐induced process but rather a biologically‐controlled one with genetic determinants dedicated to this process.

To understand this biological control, we next conducted a differential label‐free shotgun proteomics comparison using nanoLC‐tandem MS^[^ ^30^ ^]^ based on the BW‐1 annotated genome.^[^ ^31^ ^]^ We compared the abundance of mass spectrometry‐certified proteins present in the total cell fractions cultured in copper‐rich (13.9 µm) and copper‐poor conditions (0.73 µm), in which cells form and do not form copper sulfide nanoparticles, respectively. We recorded a large dataset of 306, 766 MS/MS spectra, which could point at 22, 144 trypsin‐generated peptides, and identified 1750 polypeptides. Hypothesizing that biomineralizing proteins are more abundant in cell extracts of the copper‐rich condition, we listed significantly up‐detected proteins in cells in these conditions (Table S1, Supporting Information).

**Figure 4:**
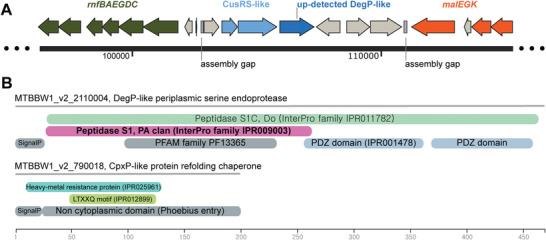
Genomic and proteomic information associated with selected up‐detected periplasmic proteins in cells producing copper sulfide particles. A) Genomic region of the up‐detected DegP‐like protein MTBBW1_v2_2 110 004. B) Domain architecture of two up‐detected putative periplasmic proteins involved in copper sulfide biomineralization.

Significant fold change was observed for two periplasmic proteins localized on different scaffolds of the genome. The one with the highest fold change (×2.2), whose sequence is found in the MicroScope database under the accession number MTBBW1_v2_2 110 004, is detected by 31 unambiguous peptides. It is homologous to the periplasmic serine endoprotease DegP and represents up to 0.19% of the total detected proteome. In the BW‐1 genome, the corresponding gene is adjacent to genes coding for a classical membrane‐bound histidine kinase and a cytoplasmic response regulator. This organization is highly similar to the two‐component (TC) CusS/CusR regulatory system involved in copper homeostasis^[^ ^32^ ^]^ (**Figure** **4**A). In addition, this region is flanked downstream by an inner membrane metabolite transporter, a NAD‐dependent epimerase/dehydratase, a NAD(P)/FAD‐dependent oxidoreductase and a malEGK operon involved in maltose uptake, while a full *rnfABCDGEH* operon is located upstream. Some of these genes have iron‐sulfur centers that can participate in oxidation‐reduction processes, whose role in periplasmic biomineralization should be investigated.

The DegP‐like protein consists of a N‐terminal protease domain coupled to two PDZ domains (Figure 4B). The protease domain shares a high degree of sequence identity (48% on average) with the N‐terminal region of MamE paralogs detected in BW‐1 and *Magnetospirillum magneticum* AMB‐1 genomes. MamE belongs to the HtrA/DegP family and is required for the continued growth of magnetite crystals in the magnetotactic species *M. magneticum* AMB‐1.^[^ ^33^ ^]^ However, MTBBW1_v2_2 110 004 does not have CXXCH heme‐binding motifs required for magnetite biomineralization. Thus, if it is involved in the formation of copper sulfide particles, its activity in crystal nucleation or growth could be indirect.

Indeed, DegP proteases are more commonly known to act as chaperones and activate pathways by proteolysis of other periplasmic proteins. For example, DegP can fold or unfold a periplasmic protein, MalS.^[^ ^34^ ^]^ In *Escherichia coli*, the periplasmic endoprotease DegP is known to be involved in the Cpx envelope stress response to cope with stressful conditions.^[^ ^35^ ^]^ In this process, DegP‐dependent proteolysis of the CpxP protein lifts the inhibition of the Cpx pathway, inducing the expression of other envelope protein folding and degrading factors.

Interestingly, another up‐detected, putatively periplasmic protein (MTBBW1_v2_790018, representing 0.34% of the total proteome) is detected by 14 unambigous peptides. Its sequence comprised a CpxP‐like motif LXXTQ (Figure 4B), suggesting that it could have similar interactions with DegP homologs like the protein MTBBW1_v2_2110004. Moreover, the latter protein belongs to a metal‐binding protein family, whose members were shown to bind zinc or copper.^[^ ^36^ ^]^ Considering the functions and the interaction between two periplasmic proteins, we suggest that the heavy metal‐binding protein binds to copper and interacts with the DegP‐like protein to cleave peptide bonds in proteins in the periplasm. This process possibly contributes to the formation and encapsulation of the CuS nanocrystallites within the periplasm.

## 3 Conclusions

We propose that the biomineralization process found in the periplasm of BW‐1 involves tight biological control. From a mechanistic point of view, our proteomics data support the following scenario: sulfide ion generated by sulfate reduction and copper ion accumulated into the periplasmic space form particles by activating a specific regulatory pathway as suggested above. However, in the absence of a genetic system for this strain, no gene deletion can be performed to directly test the role of the above‐mentioned biological determinants. Future experiments, including spectroscopy, comparative genomics, transcriptomics, and directed genetic approaches with computational simulations will explain how a simple microorganism is capable of producing copper sulfide nanocrystallites and the surrounding organic substance and of stabilizing a metastable mineral phase. In addition, further tests should reveal whether the biomineralization process could constitute a strategy to detoxify the copper ions in the cell.

## 4 Experimental Section

### Cell Culture

Pre‐culture preparation: BW‐1 cells were first cultured at 28 °C in a culture medium of Cu(II) 0.73 µm and Fe(II) 1 µm. In this culture condition, the bacteria rarely produce copper‐based or iron‐based intracellular biominerals.

Cell culture preparation to produce particles: Once the pre‐culture was enriched with motile cells (cell concentration > 2 × 10^7^ cells mL^–1^), cells were centrifuged down and inoculated to a new culture medium containing enhanced concentration of Cu(II) 13.9 µm and Fe(II) 20 µm. The initial cell density was controlled below 10^6^ cells mL^–1^ to maintain the amount of extracellular precipitates produced by cellular metabolites and metal ion concentration in the medium. The cell culture was kept in the dark at 28 °C for 20 h to make cells produce copper sulfide particles.

The culture medium used for BW‐1 culture contains (per liter): 20 g NaCl, 3 g MgCl_2_·6H_2_O, 3 g Na_2_SO_4_, 0.2 g CaCl_2_·2H_2_O and 0.5 g KCl, 5 mL modified Wolfe's mineral elixir,^[^ ^37^ ^]^ 0.3 g NH_4_Cl, 4.8 g HEPES and 2 g fumaric acid. After the pH value was adjusted to 7.2 with 5 m NaOH, the medium was autoclaved. Once the medium was cooled down to room temperature, 0.5 mL of an anaerobic stock solution of vitamins,^[^ ^38^ ^]^ 1.8 mL of 0.5 m KHPO_4_ buffer (pH 7.0), 0.4 g of sterilized cysteine were added. Cu(II) and Fe(II) concentrations were adjusted by adding FeSO_4_·7H_2_O solution and CuSO_4_·5H_2_O solution according to the need for each experiment. After all additives were added, the culture bottles were closed with septum caps and purged with N_2_ for 30 min.

### Transmission Electron Microscopy

Cells were deposited on Ni grids coated with carbon film, and cells and particles imaged using a Tecnai G2 BioTWIN transmission electron microscope (FEI Company) at 100 kV acceleration voltage. The number of particles on transmission electron microscopy (TEM) images were manually counted using ImageJ software. Isolated particles were prepared in three steps:^[^ ^1^ ^]^ filtration of cells with filter papers having 8 µm pore size;^[^ ^2^ ^]^ ultracentrifugation with sucrose gradients of 50%, 60%, 70% w/w to remove precipitates found under sucrose gradient of 70% w/w and collect cells under sucrose gradient of 50% w/w and 60% w/w;^[^ ^3^ ^]^ cell disruption was carried out using a disruptor at 2000 bar. After cell disruption, particles were dyed either with 2% w/v sodium tungstate solution for 5 min or with 0.5% w/v uranyl acetate for 2 min.

For ultra‐thin sections, BW‐1 cells were fixed in a buffer prepared in the artificial seawater of the BW‐1 medium containing 2.5% w/v glutaraldehyde and 0.1 m sodium cacodylate, pH 7. Cells were kept at 4 °C for at least 24 h. Cells were then postfixed for 1 h with 1% w/v of osmium tetroxide. Cells were then dehydrated with successive ethanol baths with increasing concentrations and finally embedded in the resin (Epon 812). Sections (nominal thickness ≈ 50 nm) were made with the ultramicrotome UC7 (Leica Microsystems GmbH), deposited onto TEM copper grids coated with carbon film and stained with Uranyless and lead citrate.

### High Resolution Transmission Electron Microscopy, Scanning Transmission Electron Microscopy‐High‐Angle Annular Dark‐Field, and Energy dispersive X‐ray spectroscopy

In order to study chemical compositions and structures of the particles, a ThermoFisher Talos F200X G2 scanning transmission electron microscope (STEM) at 200 kV accelerating voltage was used. Bright‐field and HRTEM images and selected‐area electron diffraction (SAED) patterns were obtained in TEM mode and recorded on a 4k × 4k Ceta camera. In STEM mode high‐angle annular dark‐field (HAADF) images were collected with 10.5 mrad beam convergence angle. Energy dispersive X‐ray spectroscopy (EDS) mapping in STEM mode was performed with 10 µs dwell time. Electron tomography was performed in STEM mode, with HAADF images acquired at 2° specimen tilt intervals within a range of ±70°.

### Scanning X‐Ray Fluorescence Microscopy and Cu K‐Edge X‐Ray Absorption Spectroscopy

TEM grids of BW‐1 cells were mounted for measurement at the I14 hard X‐ray nanoprobe beamline (Diamond Light Source Ltd., Didcot, UK) using custom holders designed and supplied by the beamline. Scanning X‐ray fluorescence microscopy measurements were conducted under ambient conditions using an incident photon energy of 9 keV for X‐ray fluorescence (XRF) mapping. The focused X‐ray beam was measured to be ≈50–60 nm in size during the beamtime. X‐ray fluorescence from the sample was collected in front of the sample using a four‐element silicon drift detector (RaySpec, UK). A raster scanning step size of 50 nm was used for high resolution maps with a dwell time of 20 ms. To collect Cu K‐edge XAS on a single cell of BW‐1, maps of Cu K*α* XRF were collected from 8.8–9.2 keV and post‐aligned using the Ca K*α* signal of a background feature. Dawn software was used to interpret collected XRF maps and extract normalized X‐ray absorption spectroscopy (XAS spectra).^[^ ^39^ ^]^

Reference materials CuO and CuSO_4_ were prepared as pellets (using pestle and mortar, then with pellet press) and used for qualitative comparison of XAS data. XAS measurements of references (and Cu foil) were collected in transmission mode at the I14 nanoprobe using a photodiode detector. Additional references were sent by Alain Manceau for comparison to BW‐1 XAS.^[^ ^40^ ^]^ These spectra were energy calibrated to their respective Cu foil. Athena program from the Demeter package was employed to conduct XAS data normalization and energy calibration.^[^ ^41^ ^]^

### Cryo‐Electron Tomography

BW‐1 cells sample (4 µL) with 10 nm gold beads (1 µL) were applied to glow‐discharged holey carbon R2/1 Cu 200 mesh grids (Quantifoil). The grids were blotted and vitrified using a Leica EM‐GP automatic plunger under 18 °C and 90% humidity conditions. Frozen grids were kept in liquid nitrogen until used. Cryo‐ET data was collected on a Titan Krios TEM G3i (Thermo Fisher Scientific) equipped with an energy filter and a K3 direct electron detector (Gatan Inc.). Data sets were collected at 300 kV with the K3 camera (counting mode) and Volta phase plate using the Thermo Fisher Tomography software. The TEM magnification corresponded to a camera pixel size of 8.03 Å for tomograms collected at X11500 magnification and 4.54 Å for tomograms collected at X19500 magnification and (K3 counting mode), and the target defocus was set between −10 and −30 µm. The total dose for a full tilt series was about 100 electron per Å^2^. Tomogram tilt ranges were between (40° or 60°) to (−40° or −60°) with 2° to 10° steps.

### Cryo‐Electron Tomography Data Analysis and 3D Representation

The tilt series images alignment and reconstruction were performed in IMOD.^[^ ^42^ ^]^ Segmentation and 3D representation of the reconstructed tomographic data was done using Amira software (Thermo Scientific). Data segmentation was performed based on contrast variations following the unique shape and structure of each component.

### Protein Analysis with nanoLC‐Tandem MS

Cultures of 400 mL with Cu(II) 13.9 µm or with Cu(II) 0.73 µm were prepared in triplicate and inoculated from the same pre‐culture with the same number of cells, about 10^6^ cells × mL^−1^. After 20 h of cultivation, cells were harvested by centrifugation at 20 000 × *g* for 30 min at 28 °C. The supernatants were removed and the bacterial pellets consisting of about 5 mg (wet weight) of cells were quickly frozen in liquid nitrogen, and then kept at −80 °C. Proteins were extracted from bacterial pellets and subjected to a 5 min SDS‐PAGE electrophoresis as recommended.^[^ ^43^ ^]^ The whole proteome was subjected to in‐gel trypsin proteolysis and the resulting peptides were characterized by tandem mass spectrometry performed with a Q‐Exactive HF instrument (Thermo) coupled to a nanoUPLC in similar conditions as previously described.^[^ ^44^ ^]^ MS/MS spectra were interpreted against the *D. Magnetovallimortis* BW‐1 theoretical annotated proteome with Mascot Daemon software version 2.6.1 (Matrix Science), taking into consideration a parent peptide tolerance of 5 ppm and MS/MS fragment tolerance of 0.02 Da. Peptides and proteins were identified with a false‐positive rate of 1%. Comparison of protein abundance between culture conditions was performed using the TFold test on the basis of the number of MS/MS spectra assigned per protein (spectral counts) as proxy of protein abundances as recommended.^[^ ^30^ ^]^ The list of CDS used in this study for proteomics was obtained using the functional annotation performed with the MicroScope platform^[^ ^45^ ^]^ and the genome of *D. Magnetovallimortis* strain BW‐1 published by Lefèvre et al.^[^ ^31^ ^]^ (RefSeq assembly accession number GCF_900 170 035.1).

## Conflict of Interest

The authors declare no conflict of interest.

## Supporting information

Supporting Information

Supplemental Movie 1

## Data Availability

The data that support the findings of this study are available in the supplementary material of this article.
