# Supplementary material for: Periplasmic Bacterial Biomineralization of Copper Sulfide Nanoparticles
Source: Adv Sci (Weinh). 2022 Aug 17;9(28):2203444. doi: 10.1002/advs.202203444 (PMC9534983; doi:10.1002/advs.202203444)
Supplement: Supplementary file 1 — Supporting Information [file ADVS-9-2203444-s001.pdf]

Supporting Information

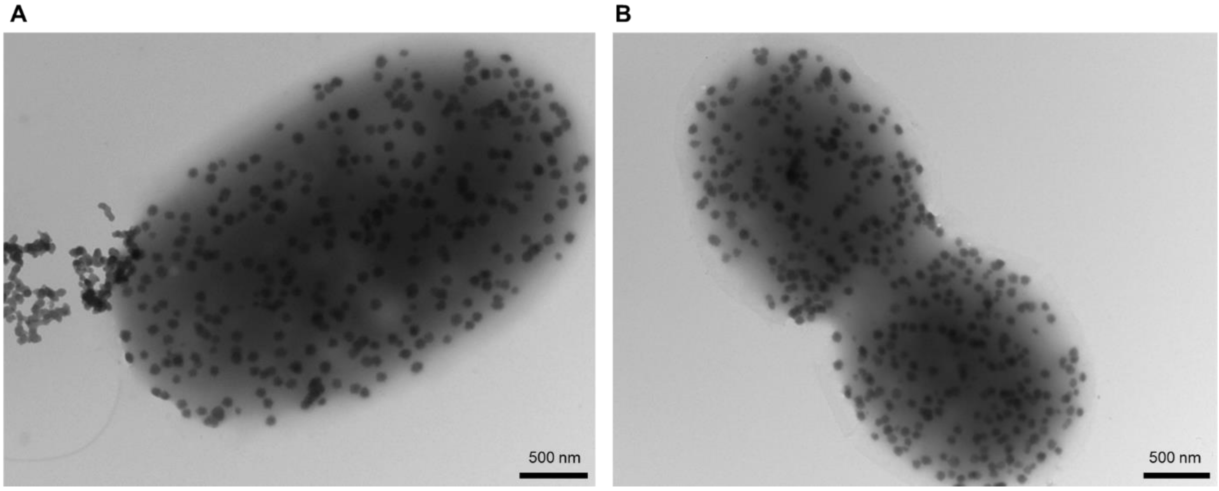

Figure S1. TEM images of BW-1 cells and biomineralized nanoparticles in 20 hours of culture. (**A**) A BW-1 cell displaying intracellular particles, next to extracellular precipitates. The intracellular particles are dispersed over the cell, whereas extracellular precipitates adhere to one another. (**B**) A BW-1 cell dividing into two daughter cells. The particles are sparsely distributed in the middle part where the cell division occurs.

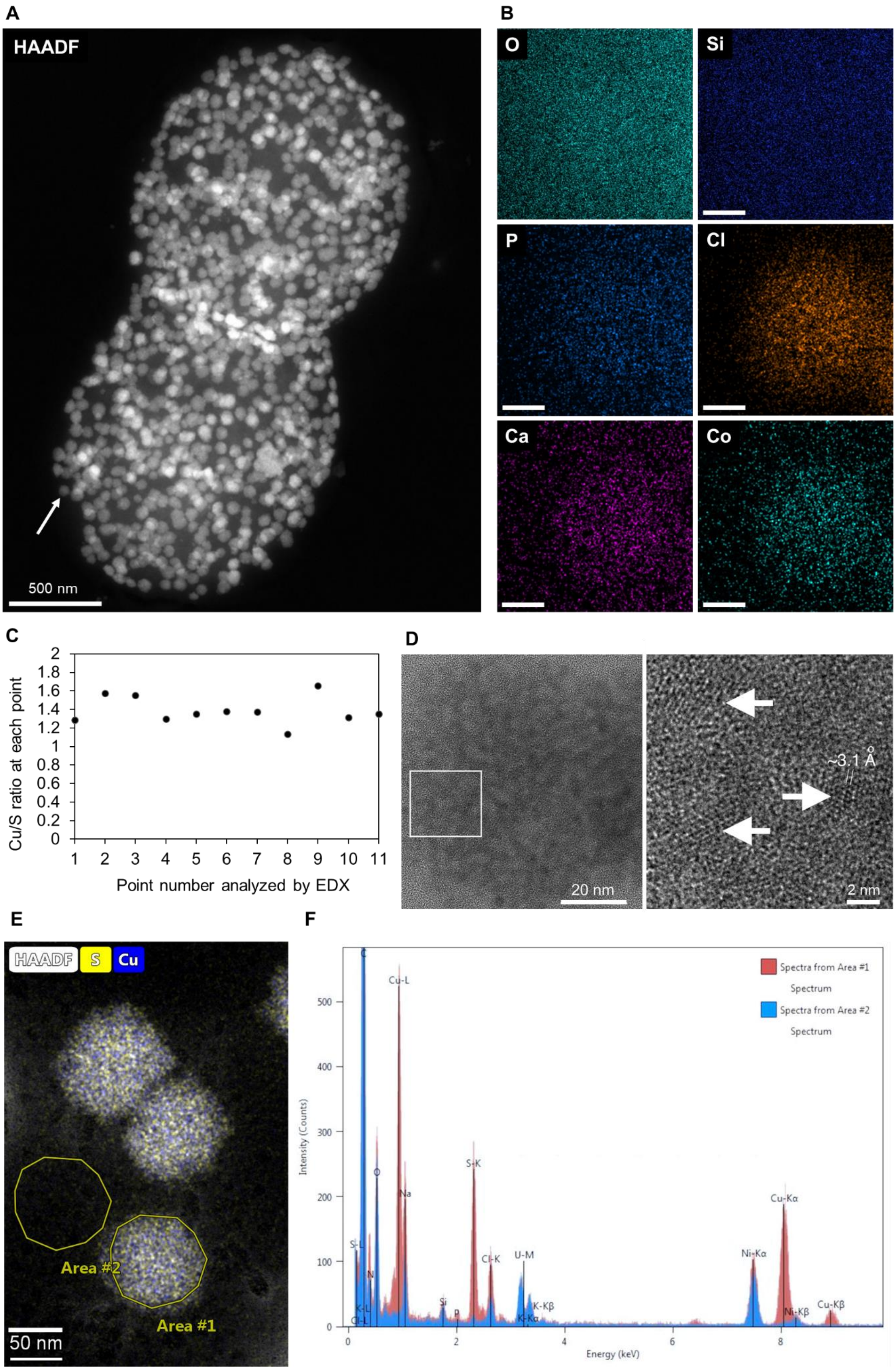
A
HAADF
500 nm
B
O
Si
P
Cl
Ca
Co
C
Cu/S ratio at each point
Point number analyzed by EDX
0 0.2 0.4 0.6 0.8 1 1.2 1.4 1.6 1.8 2
1 2 3 4 5 6 7 8 9 10 11
D
~3.1 Å
20 nm
2 nm
E
HAADF S Cu
Area #2
Area #1
50 nm
F
Spectra from Area #1 Spectrum
Spectra from Area #2 Spectrum
Intensity (Counts)
Energy (keV)
C
Cu-L
O
Na
S-K
Cl-K
U-M
Ni-Kα
Cu-Kα
Ni-Kβ
Cu-Kβ
Si
P
K-Kα
K-Kβ
Si-L
N
K-L
Cl-L

Figure S2. HAADF-STEM image and EDS analysis of a BW-1 cell with abundant intracellular copper sulfide nanoparticles. (**A**) HAADF image showing the distribution of copper sulfide nanoparticles in a dividing BW-1 cell. (**B**) STEM EDS element maps of O, Si, P, Cl, Ca, and Co, taken from a nanoparticle indicated by a white arrow in (A). Compared to Cu and S elements represented in Figure 1B, the signal intensity of the other elements is much weaker. (**C**) Cu/S ratio values obtained at 11 different points within the particle in (**B**) by EDS measurement. Average Cu/S value of the 11 points corresponds to 1.39 ± 0.15. (**D**) HRTEM image of a particle showing 1-2 nm crystallites. Individual crystallites are marked by white arrows in the right panel, which is an enlarged version of the boxed area in the left panel. (**E**) HAADF image and signals of Cu and S taken from a BW-1 cell. (**F**) EDS spectra taken from the cell in (**E**) on a part where there is a particle (#1) and no particle (#2).

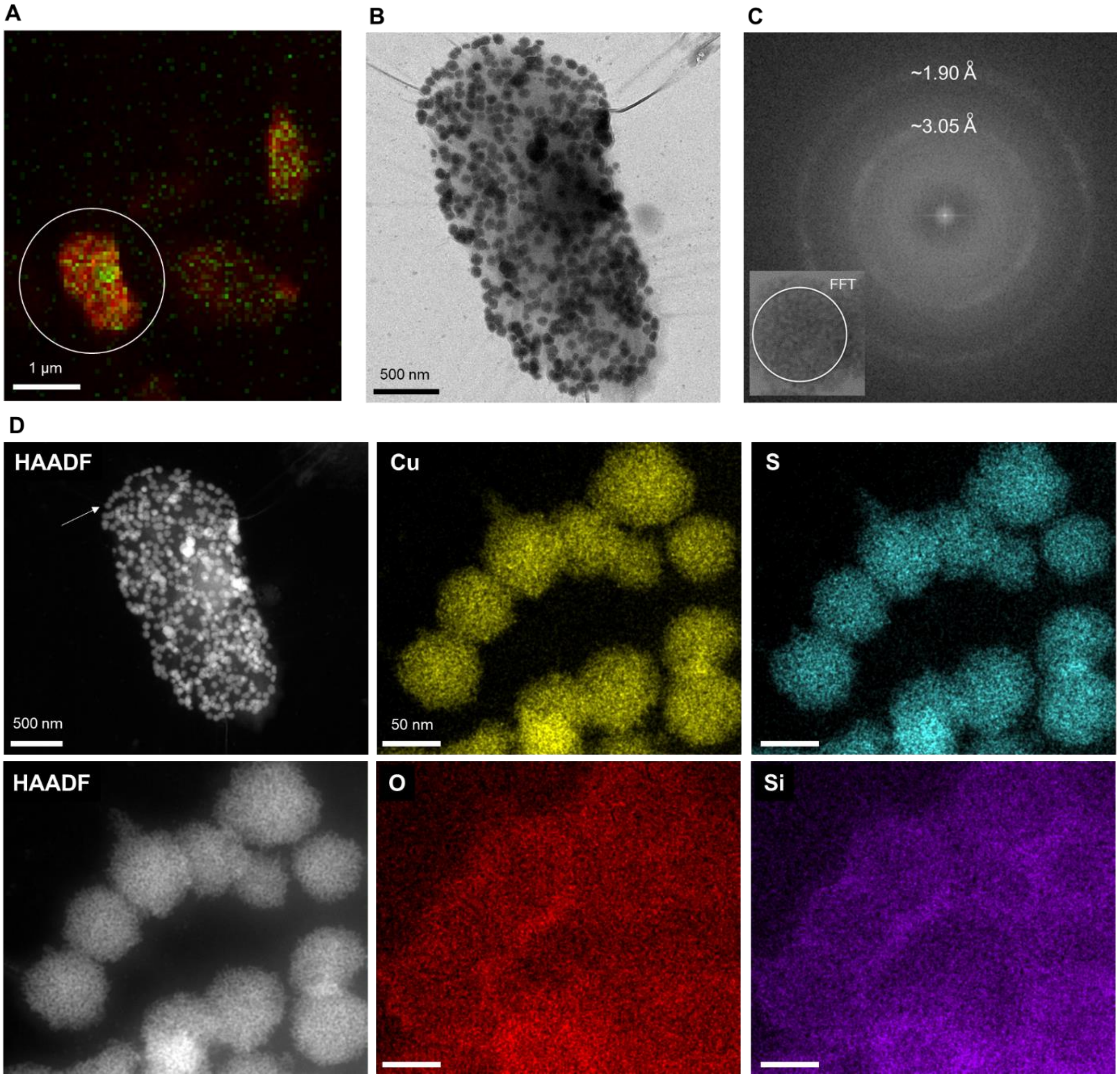

Figure S3. Structural and chemical analysis of copper sulfide nanoparticles in a BW-1 cell. (**A**) Scanning X-ray fluorescence microscopy image of the cells (Red: Cu, Green: S). (**B**) The same BW-1 cell with copper sulfide nanoparticles presented in (A) (in the white circle), imaged by TEM. The cell shrunk after a single cell XANES measurement. (**C**) Fourier transform of the boxed area of the HRTEM image in the inset, obtained from a part of the cell in (B), showing two rings and no clear spots. (**D**) HAADF images showing distribution of copper sulfide nanoparticles in the BW-1 cell in (B), and STEM EDS element maps of Cu, S, O and Si, which are taken from the area indicated by an arrow in the first HAADF image.

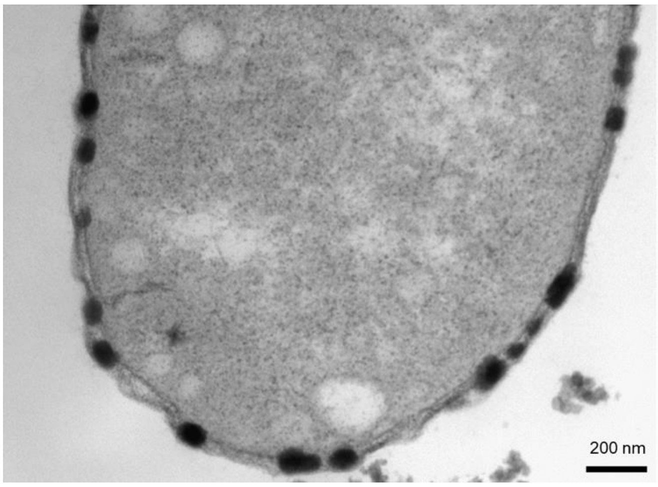

Figure S4. Transmission electron microscope image of a thin section of a BW-1 cell. Copper sulfide particles are located only within the periplasmic space. After a process of positive staining, the particles appear dark and a few of them seem to be attached to one another. The particles expand the periplasmic space, once the diameter becomes larger than the width of the periplasmic space.

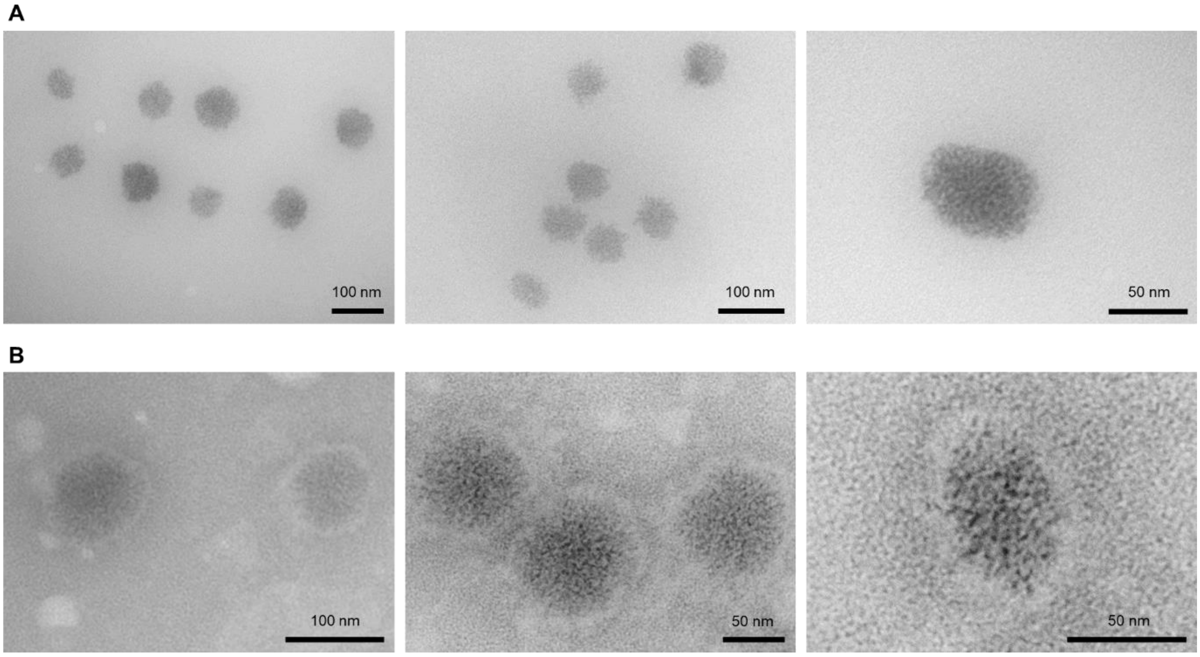

Figure S5. Copper sulfide particles deposited onto EM grid after using a cell disruptor. (**A**) Particles before negative staining. (**B**) Particles after a negative staining with sodium tungstate 2% (w v^{-1}). The particles are apparently surrounded by a macromolecular matrix.

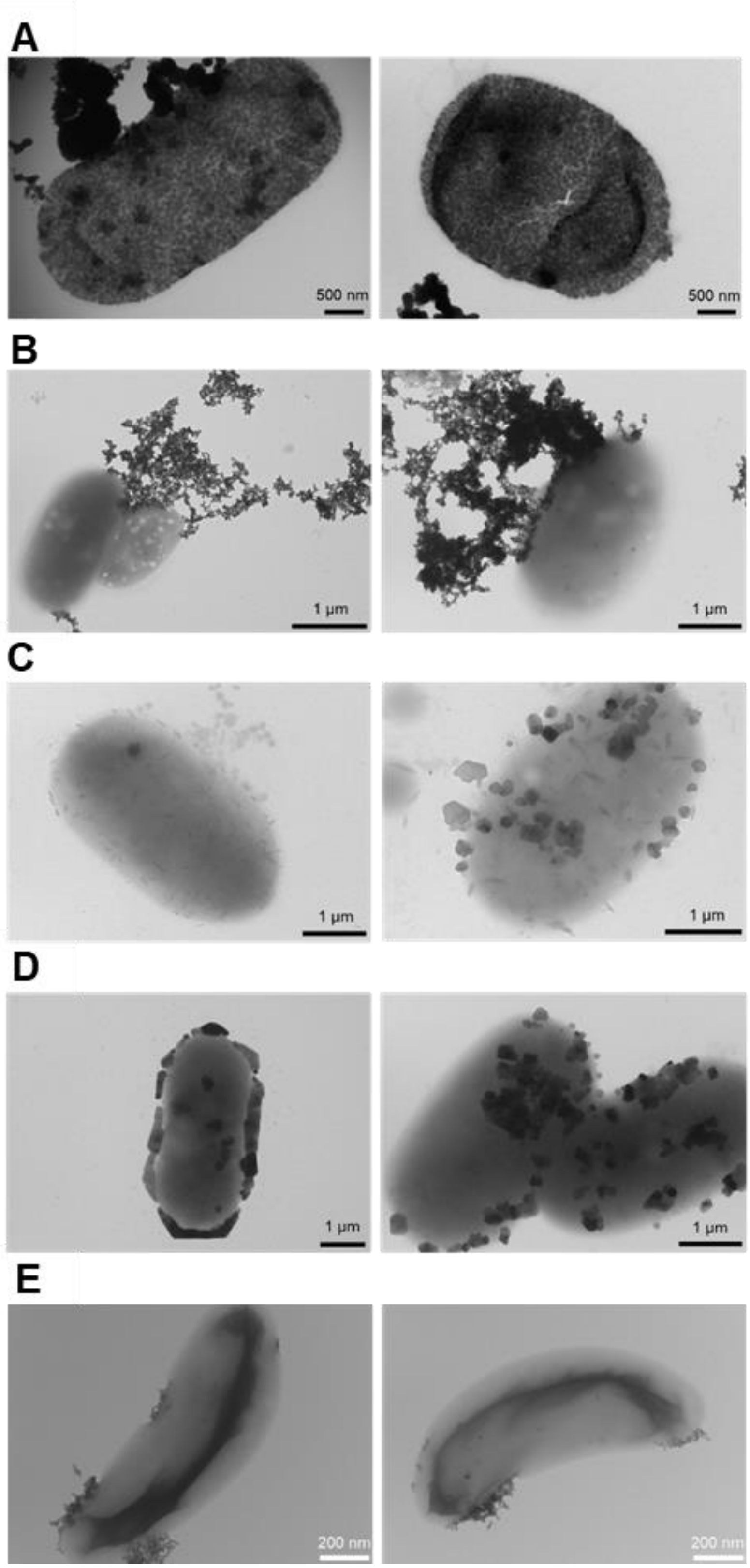

Figure S6. TEM images of BW-1 cells and RS-1 cells cultivated in the culture medium with high concentrations of several different metal ions for 20 hours. (**A)** BW-1 Cu 146 μM, (**B**) BW-1 Zn 147 μM, (**C**) BW-1 Ni 147 μM, (**D)** BW-1 Co 147 μM, respectively. (**E**) RS-1 Cu 13.9 μM. No notable intracellular particle formation was observed inside the BW-1 cells apart from A.

Table S1. Up-detected proteins in BW-1 producing copper sulfide particles (Fold change ≥ 1.5, P value ≤ 0.05)

Proteins in bold letters are presented in the Figure 4 as a pair of responsible proteins for intracellular copper sulfide biomineralization.

PB id	Accession (MTBBW1_v2_ is omitted at the beginning)	Blast functional annotation	Fold change	p value	Secondary structure best hit (function, e-value)
Heavy metal resistance-associated proteins					
A0A1W1HKI1	830056	Unknown	2.50	1.3E-02	Zinc resistance-associated protein (3LAY_F, 90.84, 1.3)
**A0A1W1HJH8**	**790018**	**Unknown**	**1.65**	**3.5E-03**	**Putative periplasmic zinc resistance-associated protein (3LAY_F, 99.71, 5.4e-15)**
Proteins found encoded with interesting cistrons/genes in the same putative operon					
**A0A1W1HCI0**	**2110004**	**Putative periplasmic serine endoprotease DegP-like**	**2.24**	**2.5E-03**	**Protease Do, protein quality control, serine protease (1KY9_A, 100, 1.5e-37)**
A0A1W1HFG7	290015	Unknown	1.70	7.8E-03	DsrE/DsrF-like family protein (3MC3_A, 99.87, 1.4e-20)
Putative cofactor biosynthesis proteins					
A0A1W1HHS5	530001	Radical SAM domain protein	2.75	7.8E-03	Oxidoreductase, coenzyme PQQ synthesis protein, peptide modifying enzyme (6C8V_A, 99.97, 3e-28)
A0A1W1HE02	2340003	Radical SAM additional 4Fe4S-binding domain-containing protein	2.67	3.8E-02	Oxidoreductase, coenzyme PQQ synthesis protein, peptide modifying enzyme (6C8V_A, 99.97, 3e-28)
A0A1W1HGN6	350033	moeA MoeA2	1.92	3.3E-02	Molybdopterin cofactor biosynthesis (1UZS_A, 100, 5.4e-66)
A0A1W1HIG7	600076	Putative sirohydrochlorin cobaltochelatase	1.71	3.8E-02	Sirohydrochlorin ferrochelatase, chelatase, biosynthetic protein
Cytoplasmic membrane proteins					
A0A1W1H7F4	1310106	pspA Regulatory protein for phage-shock-protein operon	2.44	6.1E-03	Hypothetical ATP-binding protien, AAA+ ATPase (2FNA_A, 99.13, 4.4e-9)
A0A1W1HFZ3	300087	Unknown	2.20	1.3E-02	Uncharacterized membrane protein, coiled coil, beta-layer (6H9L_A, 97.79, 0.0017)
A0A1W1HIC6	60028	Predicted ABC-type transport system, membrane protein	1.62	2.0E-02	Intraflagellar transport protein S2 homolog, transport protein (SFMS_C, 99.83, 9.5e-20)
Other upregulated proteins					
A0A1W1H5W5	1110019	infA Translation initiation factor IF-1	2.20	1.3E-02	Translation initiation factor IF-1 (6C00_A, 99.72, 4.6e-16)
A0A1W1HDE1	2270007	pyrR Pyrimidine operon regulatory protein, Uracil phosphoribosyltransferase	2.17	3.6E-02	Ancestral PyrR protein, RNA binding protein (4P82_A, 99.92, 3.3e-22)
A0A1W1HFV2	2930002	HicB-like domain-containing protein	2.00	2.4E-02	Antitoxin HicB (6U0I_C, 99.78, 4.6e-16)
A0A1W1H573	1040049	AAA domain-containing protein	2.00	4.8E-02	Walker-type ATPase (2QEN_A, 99.96, 8.1e-25)
A0A1W1H5P5	1110033	rpmC 50S Ribosomal subunit protein L29	1.92	10.0E-03	50S ribosomal protein L29 (1R73_A, 99.84, 5.3e-20)
A0A1W1H574	1050003	Unknown (Amidinotrasnferase)	1.91	1.3E-02	Arginine-guanidine removing enzyme, arginine dihydrolase (6LRF_B, 100, 2.7e-34)
A0A1W1H683	1200021	Unknown (RNA-binding, UPF0109 protein)	1.88	3.6E-02	Cag-alfa, ATPase, hydrolase, protein-Protein complex (2PT7_H, 99.66, 5.2e-16)
A0A1W1HIV6	640014	Dbi Acyl-CoA-binding protein homolog	1.64	3.1E-03	Acyl-CoA-binding domain-containing protein, lipid binding (3FLV_B, 99.85, 4.8e-20)

A0A1W 1H9D9	1650 020	rny Ribonuclease Y	1.58	3.6E -02	Phosphodiesterase, Hydrolase (3TM8_A, 99.33, 1.4e-11)
A0A1W 1HCK9	2130 084	Roadblock/LC7 family protein	1.57	4.8E -02	Gliding protein MglB, GTPase activating protein, bacterial polarity (3T1S_A, 99.88, 4.5e-20)
A0A1W 1HJI8	7900 04	Hydrolase, peptidase M42 family	1.57	4.8E -02	Deblocking aminopeptidase (2GRE_C, 100, 2.4e-30)

Movie S1. Cryo-EM tomogram showing copper sulfide nanoparticles and four cellular membranes in BW-1. (Four layers starting from outward to inward are S-layer, outer membrane, peptidoglycan layer, and plasma membrane). The nanoparticles are located in the periplasmic space.
